# The current distribution and characterization of the *L1014F* resistance allele of the *kdr* gene in three malaria vectors (*Anopheles gambiae, Anopheles coluzzii, Anopheles arabiensis*) in Benin (West Africa)

**DOI:** 10.1186/s12936-019-2808-9

**Published:** 2019-05-21

**Authors:** Come Z. Koukpo, Arsène Jacques Y. H. Fassinou, Razaki A. Ossè, Fiacre R. Agossa, Arthur Sovi, Wilfrid T. Sewadé, Sidick Aboubakar, Bénoît S. Assogba, Martin C. Akogbeto, Michel Sezonlin

**Affiliations:** 1 Cotonou Entomological Research Centre (CREC), Cotonou, Benin; 2 Laboratory Evolution, Biodiversity of Arthropods and Sanitation, FAST-UAC, Abomey-Calavi, Benin; 3 School Doctoral Sciences of Life and Earth, FAST-UAC, Abomey-Calavi, Benin; 4 Ecole de Gestion Et D’Exploitation Des Systèmes D’Elevage, Université Nationale D’Agriculture, Kétou, Benin; 5 Regional Institute of Public Health, University of Abomey Calavi, 01BP918 Abomey Calavi, Benin

**Keywords:** Malaria, *kdr*, Gene flow, *An. gambiae*, Pyrethroids, Benin, West Africa

## Abstract

**Background:**

The fight against malaria faces various biological obstacles, including the resistance of parasites to anti-malarial drugs and the resistance of mosquito vectors to insecticides. The resistance of *Anopheles gambiae *sensu lato (s.l.) to pyrethroids, the only class of insecticides used to impregnate mosquito nets, is known in Benin; the expansion of this resistance is influenced by the existence of gene flow between species, otherwise by the presence or absence of the *kdr* mutation in them. The objective of this study is to determine the spatial distribution of *An. gambiae* and the level of expression of the pyrethroid resistance *kdr* gene in seven agro-ecological zones of Benin.

**Methods:**

The study was conducted in 18 localities belonging to seven agro-ecological zones where environmental parameters varied. The sites represent the main areas of eco-epidemiological malaria in Benin. *Anopheles gambiae* larvae were collected in natural breeding sites using ladles and dipping method and reared under standard conditions. These larvae were reared under standard conditions of temperature and humidity (26 to 30 °C and 60 to 90%) at the insectarium of the Centre de Recherche Entomologique de Cotonou (CREC). Adult female mosquitoes having emerged are morphologically and molecularly identified. Homozygous resistant (*1014F/1014F*), homozygous sensitive (*1014L/1014L*) and heterozygous (*1014F/1014L*) genotypes of the *L1014F* *kdr* gene mutation are determined by PCR.

**Results:**

A total of 677 *An. gambiae* was subjected at the PCR. The results revealed the presence of three vector species of the *An. gambiae* complex, of which 409 *Anopheles coluzzii*, 259 *An.* *gambiae*, 5 hybrids (*An. coluzzii*/*An. gambiae*) and 4 *Anopheles arabiensis* in the different agro-ecological zones. The four *An. arabiensis* were only found in Dassa, a locality in the cotton zone of central Benin. The frequency of distribution of the *L1014F* allele of the *kdr* gene varies from 84.48 to 100% in *An. gambiae*, from 80 to 100% in *An. coluzzii* and from 0 to 75% in *An. arabiensis* in the different agro-ecological zones. Moreover, a significant difference is generally observed in the distribution of the *L1014F* allele (P < 0.05). By comparing in pairs the distribution frequencies of this allele in the two species by agro-ecological zone, only a significant difference is noted in the central cotton and fishery zones (P = 0.0496).

**Conclusion:**

In summary, even if the data are in small portions, the *An*. *Arabiensis* species was found only in central Benin and the *L1014F* allele of the *kdr* gene is widespread and seems to fix in all the species recorded in the different agro-ecological zones. This situation amplifies the problem of resistance, which could eventually be a significant obstacle for the malaria vectors control. Similarly, a study of their genetic structure via the *L1014F* allele is necessary in order to put in place strategies to manage this resistance. These strategies will take into account both the ecology and the genetic diversity of the organisms involved to preserve the effectiveness of pyrethroids, the only insecticides used for the impregnation of mosquito nets.

## Background

The malaria control faces various biological obstacles, including the resistance of parasites to anti-malarial drugs [1] and of mosquito vectors to insecticides [2]. So far, the fight against this disease is essentially based on chemotherapy targeting the parasite in humans using anti-malarial drugs, as well as actions aiming at simultaneously reducing the human-vector contact, the density and the longevity of *Anopheles* vectors [2]. As a result of years of efforts to control or even eradicate malaria, it still remains a global public health problem [3].

In the absence of a vaccine to immunize humans against harmful actions of the pathogen, vector control appears for the moment as the best preventive tool [3]. Among the different methods that are commonly used are long-lasting insecticidal nets (LLINs) and indoor residual spraying (IRS) [4]. With the increased use of insecticides from different classes to combat mosquitoes effectively, vector populations resistant to these chemicals have become increasingly important [5]. The rationales are to be found in the maintenance of ancestral polymorphism and the vertiginous selection of resistance alleles that are, the most advantageous in the new environment created by the misuse of these insecticides. Indeed, several cases of emergence of vector resistance to insecticides have been described in Eastern [6] and Western [7–10] Africa.

Studies carried out in Burkina Faso [11], Benin [12] and Côte d'Ivoire [13] have shown that the frequency of the *L1014F* resistance allele of the *kdr* gene is higher in *Anopheles gambiae *sensu lato (s.l.) in agricultural areas usually treated with insecticides, as compared to rural areas where farmers grow only food or local consumption products. The hypothesis of the contribution of certain agricultural practices to the selection and extension of resistance in vectors cannot be excluded. In the current context where pyrethroids resistance is very widespread in Africa [2], studies on the diversity of this insecticide resistance gene in vector populations are important to improve the control tools used against *An. gambiae,* major vector of malaria.

Globally, the larvae of *An. gambiae* grow in small, shallow, relatively clean and sunny water reservoirs (puddles of water, stagnant water) [14]. The increasing presence of *An. gambiae* s.l. in ecological sites, which were originally inappropriate and unsuitable for this species [15], leads to believe that this species has developed mechanisms that allow it to adapt to xenobiotics present in these new environments, the said mechanisms being characteristic of the normal biological evolution of these vectors. Clearly, the link between resistance and these new adaptations is still not formally established. Benin is a country of great geographical (climatic, relief) and ecological variability [16]. It is, therefore, imperative to develop more molecular studies on the vectors that are encountered in order to propose more effective and sustainable control methods that take into account the genetic diversity within the *An. gambiae* complex according to the different ecological and geographical niches. In addition to the fact that the resistance of *An. gambiae* to pyrethroids, the only class of insecticides used for the impregnation of mosquito nets [7–9], is known and well documented in Benin [7–9], the expansion of this genetic and hereditary trait is strongly influenced by the existence of gene flows maintained through migration, preferential mating or inbreeding between different populations of the same species. In view of the dynamics that characterize populations of living organisms, it is important to focus on the biological evolution in time and space of *An. gambiae *sensu stricto (s.s.)*, An. coluzzii* and *An. arabiensis*, three sibling species taken into account in the current study. Thus, we will to study the spatial distribution of these three species and of the resistance allele *L1014F* of the *kdr* gene within them in seven agro-ecological zones in order to better orient the control methods.

## Methods

### Characteristics of the study area

**Fig. 1:**
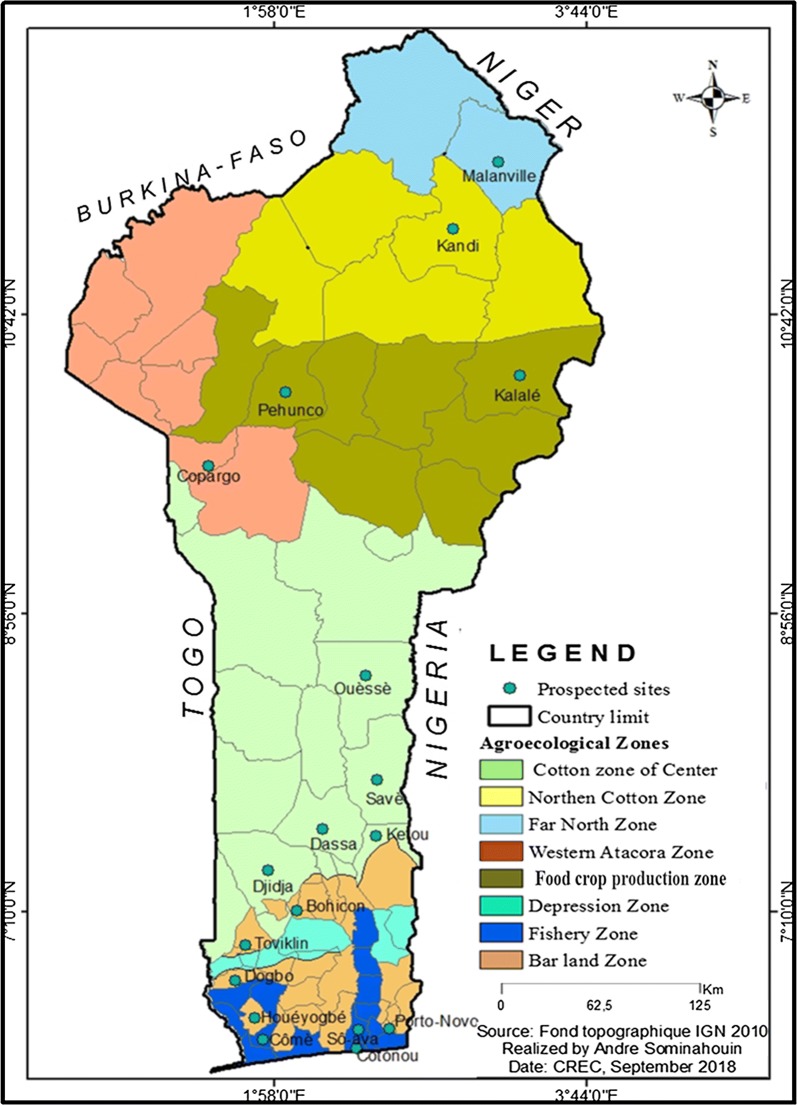
Map of the different agro-ecological zones of Benin with the communes of studies

The current study was implemented in Benin, a West African country located in the intertropical zone between the equator and the Tropic of Cancer, more precisely between the parallels 6°30′and 12°30′ of latitude North on the one hand, and the meridian 1° and 3°40′ East longitude on the other hand. Benin has a subequatorial climate with four seasons (two rainy seasons interspersed by two dry seasons) in the south; a Sudano-Guinean transitional climate with four seasons similar to that of the South in the Centre of the country and, a Sudanese climate with two seasons (a rainy and a dry) in the north [17]. The relief of Benin is not very rugged but has a low coastal and sandy region limited by lagoons. It also presents a plateau of ferruginous clay, a silico-clay plateau dotted with some undergrowth [17]. In the north-west of the country is the massive Atacora with a height of 800 m and in the north-east, the silico–clay fertile plains of Niger [17]. Benin has a large hydrographic network with two important basins namely: the Niger basin with several tributaries and the coastal basin whose rivers (Ouémé, Zou, Mono) reach the Atlantic Ocean. Each basin is dotted with small permanent or temporary water bodies that maintain agro-pastoral activities [17]. Overall, eight agro-ecological zones have been defined in Benin [16], namely: the far north zone of Benin (Zone 1), the northern cotton zone of Benin (Zone 2), the food crop production zone of South-Borgou (Zone 3), Western Atacora Zone (Zone 4), Cotton Zone of Centre (Zone 5), bar land Zone (Zone 6), Depression Zone (Zone 7) and Fisheries Zone (Zone 8). Seven of the eight Zones are subject to this study. They are classified on the basis of homogeneity taking into account the consideration of climatic and agro-pedological parameters, cropping systems, population density and vegetation cover [16]. In total, eighteen study sites were selected from seven different agro-ecological zones (Fig. 1). Thus, the districts of Dogbo, Houéyogbé, Toviklin, Bohicon and Porto-Novo have been selected in the bar land Zone. Those of Dassa, Savè, Ouèssè, Djidja and Kétou were selected in the cotton zone of central Benin. The districts of Malanville, Kandi and Copargo are, respectively, located in the zones of the far north, Northern cotton and Western Atacora. The districts of Kalalé and Péhunco are considered as being in the food crop production zone of South Borgou, while those of Cotonou, Comè and Sô-Ava are in the fishery zone.

### Sampling and treatment of mosquitoes

#### Larvae collections and mosquito rearing

Larvae collections were conducted in all study sites from May 2015 to December 2017. They are made only during rainy seasons. Larvae of *An. gambiae* were collected from the positive breeding sites encountered using the dipping method. The latter consists in collecting mosquito larvae from the surface of the breeding sites through a ladle. The collected larvae were filtered and kept in labeled jars according to the surveyed sites and then transported to the insectary of the Center for Entomological Research of Cotonou (CREC). These larvae were raised under standard conditions of temperature (27 °C ± 2) and humidity (65% ± 10) until adult stage.

### Molecular identification and species distribution

Adult female mosquitoes resulting from reared larvae were morphologically identified using morphological identification keys of Gillies and Coetzee [18] and of, Gillies and De Meillon [19]. They are then subjected to the polymerase chain reaction (PCR). Thus, at least 30 mosquitoes were analysed by site. The SINE 200 PCR of Santolamazza et al. [20] and the PCR protocol of Scott et al. [21] were carried out for the identification of the species of the *An. gambiae* complex.

### Determination of the *L1014F* mutation of the *kdr* gene in the three species

The resistant allele *L1014F* of the *kdr* gene was determined according to the protocol of Martinez-Torres et al. [22]. This PCR diagnostic test consists in using four oligonucleotides or primers (Agd1, Agd2, Agd3, Agd4) and a Taq polymerase to search by amplification for resistant or susceptible alleles on a DNA fragment coding for the voltage-dependent sodium channel in each tested mosquito. The Agd1/Agd2 primers pair flanks the *kdr* gene by amplifying a 293 bp product as a control. The pair of Agd3/Agd1 primer pairs only with the resistance portion of the *kdr* gene to amplify a 195pb fragment. The Agd4 /Agd2 pair associates only with the portion of the sensitive gene by amplifying a 137 bp fragment. According to the protocol of Martinez-Torres et al. [22], the nucleotide sequences of these primers are: Agd1: 5′-ATAGATTCCCCGACCATG-3′; Agd2: 5′-ACAAGGATGATGAACC-3′; Agd3: 5′-AATTTGCATTACTTACGACA-3′; Agd4: 5′-CTGTAGTGATAGGAAATTTA-3'.

### Analysis of the geographical distribution of the *L1014F* allele of the *kdr* gene in *Anopheles gambiae*, *Anopheles coluzzii* and *Anopheles arabiensis*

The frequencies of the *L1014F* allele of the *kdr* gene were determined by agro-ecological zone. They were calculated using the F-statistics formula [23] of the Genepop software version 4.2. The confidence intervals of the allelic frequencies were calculated using the binomial formula. The proportion test [24] of the R software, version 3.3.3 was used to compare the distribution of the different species and the of *L1014F* allele frequencies within these species of the various agro-ecological zones. It was therefore assessed whether the species and their resistance allele are distributed in the same way in the different zones.

## Results

### Molecular identification and distribution of the different species

Table 1 provides results obtained from the molecular identification of species. After molecular species identification performed on *An.Gambiae,* the presence of three sibling vector species was revealed. They were distributed in variable proportions with a total of 409 *An. coluzzii* (60.41% ± 3.68), 259 *An. gambiae *s.s. (38.26% ± 3.66), 05 hybrids ((0.74% ± 0.64) *An. coluzzii*/*An. gambiae*) and 04 *An. arabiensis* (0.59% ± 0.58).

**Table 1:** Distribution frequency and number of *Anopheles gambiae*, *Anopheles coluzzii* and *Anopheles arabiensis* in the different agro-ecological zones

Agro-ecological zones	Localities	Geographical coordinates	Number tested	*Anopheles gambiae* N (%, IC)	*Anopheles coluzzii* N (%, IC)	*Hybrides forms* N (%, IC)	*Anopheles arabiensis* N (%, IC)	*p-*values
Far north zone	Malanville	11°51′48″N 3°23′3,29″E	30	0	30	0	0	
	Total		30	0 (0%)	30 (100%)	0 (0%)	0 (0%)	–
Northern cotton zone	Kandi	11°7′49,869″N 2°55′57,282″E	30	29	1	0	0	
	Total		30	29 (96.67 ± 6.42)	1 (3.33 ± 6.42)	0	0	< 0.001
South-Borgou food crop production zone	Kalalé	10°17′24,469″N 3°22′54,215″E	30	30	0	0	0	
	Péhunco	10°14′13,431″N 2°0′10,209″E	30	28	2	0	0	
	Total		60	58 (96.67 ± 4.54)	2 (96.67 ± 4.54)	0 (0%)	0 (0%)	< 0.001
Western Atacora zone	Copargo	9°50′22,8″N 1°32′50,995″E	31	27	4	0	0	
	Total		31	27 (87.10 ± 11.80)	4 (12.90 ± 11.80)	0 (0%)	0 (0%)	< 0.001
Central cotton zone	Dassa	7°47′5,671″N 2°11′674″E	30	18	7	1	4	
	Savè	8°2′1″N 2°29′6″E	30	11	19	0	0	
	Ouèssè	8°29′465″N 2°25′24,03″E	30	23	7	0	0	
	Kétou	7°21′37″N 2°36′14″E	30	8	22	0	0	
	Djidja	7°20′46″N 1°56′8″E	30	2	28	0	0	
	Total		150	62 (41.33 ± 7.88)	83 (55.33 ± 7.96)	1 (0.67 ± 1.30)	4 (2.67 ± 2.58)	< 0.001
Bar land zone	Bohicon	7°10′48″N 2°5′12″E	39	35	4	0	0	
	Dogbo	6°48′17″N 1°47′17″E	39	12	27	0	0	
	Toviklin	6°56′20,73″N 1°46′24,083″E	30	6	24	0	0	
	Houéyogbé	6°31′55,83″N 1°52′15,1″E	30	18	12	0	0	
	Porto-Novo	6°29′47″N 2°37′44″E	54	4	47	3	0	
	Total		192	75 (39.06 ± 6.90)	114 (59.38 ± 6.94)	3 (1.56 ± 1.75)	0 (0%)	< 0.001
Fishery zone	Cotonou	6°21′54″N 2°27′53″E	102	2	99	1	0	
	Sô-Ava	6°27′57,687″N 2°24′4,004″E	50	0	50	0	0	
	Comè	6°24′34,62″N 1°53′7,82″E	32	6	26	0	0	
	Total		184	8 (4.35 ± 2.95)	175 (95.11 ± 3.12)	1 (0.54 ± 1.06)	0 (0%)	< 0.001
Total			677	259 (38.26 ± 3.66)	409 (60.41 ± 3.68)	5 (0.74 ± 0.64)	4 (0.59 ± 0.58)	< 0.001

Overall, the frequency of distribution ranged from 0 to 96.67% in *An. gambiae *s.s., from 3.33 to 100% in *An. coluzzii*, and from 0 to 2.67% for *An. arabiensis* according to the agro-ecological zones.

*Anopheles gambiae* was found in the proportions of 96.67% in the cotton zone of north and food crop production zone of South Borgou and, 87.10%, 41.33%, 4.35% and 39.06% respectively in the Western Atacora, Central Cotton, bar land and fisheries zones. The distribution frequencies of *An. coluzzii*, was 3.33% in the Northern cotton and food crop production zones of South Borgou, 100%, 12.90%, 55.33%, 59.38% and 95.11% respectively in the Far North, Western Atacora, central cotton, bar land and fisheries zones. *Anopheles arabiensis* was absent in the different surveyed zones except for of the central cotton zone where it was 2.67%. A significant difference is noted in the distribution of sibling species within different agro-ecological zones (p < 0.001).

*Anopheles gambiae* and *An. coluzzii* live in sympatry in six zones except the northern extreme zone. *Anopheles gambiae* is widespread in northern Benin while, *An. coluzzii* was the major species from Central to South. It is observed that the distribution is more or less homogeneous with characteristic ecological preferences. Thus, *An. gambiae* is more adapted to the ecology of the North with high frequencies in the zones of cotton production of North (96.67% ± 6.42), of food crop production of South Borgou (96.67% ± 4.54) and Western Atacora (87.10% ± 11.80). *Anopheles coluzzii* prefers the southern zones with high frequencies in the central cotton (55.33% ± 7.96), bar land (59.38% ± 6.94) and fisheries (95.11% ± 3.12) zones even though we noted its exclusive presence in the far north. *Anopheles arabiensis* was found only in Dassa, the central cotton zone, but with a low number, which may be an ecological indicator.

### Distribution of the frequency of the *L1014F kdr* mutation in *An. coluzzii*, *An. gambiae* and *An. arabiensis*

Table 2 presents the frequency distribution of the *L1014F* resistant allele of the *kdr* gene in *An. coluzzii* and *An. gambiae *s.s. in the different surveyed agro-ecological zones. Overall, the frequency of the *L1014F* resistant allele of the *kdr* gene ranged from 84.48 to 100% in *An. gambiae* and from 80 to 100% in *An. coluzzii*.

**Table 2:** Distribution frequency of the *L1014F* resistant allele of the *Kdr* gene F (*L1014F*) in the *An.Coluzzii* and *An.Gambiae* species

Agro-ecological zones	N_T_	N	*An. gambiae*				*An. coluzzii*				P-value
			N_1_ *1014F/*	N_2_ *1014F/*	N_3_ *1014L/*	F*(L1014F)%*	N_1_ *1014F/*	N_2_ *1014F/*	N_3_ *1014L/*	F*(L1014F)%*	
			*1014F*	*1014L*	*1014L*		*1014F*	*1014L*	*1014L*		
Far north zone	30	0	0	0	0	0	21	6	3	80 ± 10.12	-
Northern cotton zone	30	29	23	3	3	84.48 ± 9.32	1	0	0	100	1
South-Borgou food crop production zone	60	58	50	6	2	91.38 ± 5.11	2	0	0	100	1
Western Atacora zone	31	27	25	2	0	96.30 ± 5.04	3	1	0	87.5 ± 22.92	0.842
Central cotton zone	150	62	54	8	0	93.55 ± 4.32	65	12	6	85.54 ± 5.35	0.0496
Bar land zone	192	75	74	1	0	99.33 ± 1.30	89	22	3	87.72 ± 4.26	< 0.001
Fishery zone	184	8	8	0	0	100	140	31	4	88.86 ± 3.30	0.3181
Total	677	259	234	20	5	94.21 ± 2.01	321	72	16	87.29 ± 2.28	< 0.001

F (*L1014F*)%: frequency of the *L1014F* allele of the *Kdr* gene of the different species; NT: cumulative total number of species by agro - ecological zone; N: represents the number of each species; N1: number of homozygous resistant genotype *1014F/1014F*; N2: the heterozygous genotype *1014F/1014L*; N3: number of susceptible homozygous genotype *1014L/1014L*; *1014F/1014F*: resistant homozygous genotype; *1014F/1014L*: heterozygous genotype; *1014L/1014L*: susceptible homozygous genotype

In *An. gambiae*, the frequencies were 0%, 84.48%, 91.38%, 96.30%, 93.55%, 99.33%, and 100% respectively in the Far North, Northern Cotton, food crop producing of South Borgou, West-Atacora, Central Cotton, laterite and Fisheries zones.

In *An. coluzzii*, the frequencies were 100% in the northern cotton and food crop production zones of South Borgou, and 80%, 87.5%, 85.54%, 87.72%, 88.86% and 87.29% respectively in the far north, western Atacora, central cotton, bar land and fisheries zones. On the other hand, a significant difference is generally observed in the distribution of the *L1014F* allele (p < 0.05). The frequencies of the *L1014F* allele of the *kdr* gene are similar between *An. gambiae* and *An. coluzzii* in all agro-ecological zones except for the central cotton (p = 0.0496) and of the fishery (p < 0.001) zones. The small number (n = 4) of *An. arabiensis* detected did not allow a valid comparison of the frequency of the *L1014F* allele of the *kdr* gene of this species with the one of *An. coluzzii* and *An. gambiae*.

Table 2 shows that the frequency of the *L1014F* allele of the *kdr* gene is at least 80% ± 10.12 in the different species of the different agro-ecological zones. In the same agro-ecological zone, there is no significant difference (p > 0.05) in the distribution of this allele in the two species except in the central cotton (p = 0.0496) and bar land zones (p < 0.001). Similarly, within the agro-ecological zones of the northern (far North, Western Atacora, North cotton and food crop production of South Borgou zones), no significant difference is noted in the distribution of this allele among the different species (p > 0.05). On the other hand, in the southern part of Benin (central cotton and bar land zones), there is a significant difference in the distribution of this allele among the different species (p < 0.05) except for the fishery zone (p = 0.3181).

## Discussion

The results of this study showed an heterogeneous distribution of *An. gambiae* and *An. coluzzii* in the different geographical areas of Benin and a strong sympatry between these two species in some localities. The presence of hybrids (*An. gambiae* /*An. coluzzii*) recorded in some study areas corroborates the results previously obtained by several authors [25–27] and supports the hypothesis of gene flow between the two species despite the well advanced speciation. The presence of hybrid suggests incomplete reproductive isolation between *An. gambiae* and *An. coluzzii* [28]. On the other hand, the fertility and the reproductive capacity of these hybrids remain to be proven since their number is small.

Overall, the results show that *An. coluzzii* was the predominant species in the southern regions of the country while *An. gambiae* was the major species in the northern and central regions. This distribution of species could be due to variable climatic conditions (a subequatorial climate with 4-seasons, 2 rainy seasons interspersed with two dry seasons in the south; a Sudano-Guinean transition climate with four seasons similar to that of the South at the center and, a Sudanese climate with a wet and a dry season in the north [17]) as well as with the ecological characteristics (physico-chemical properties of the mosquito breeding sites) of the selected areas and the sampling periods depending on the dry or rainy season. According to Mbida et al. [29], *An. coluzzii* is associated to permanent breeding sites and those resulting from human activity, while *An. gambiae* is more frequent in rain-dependent temporary breeding sites. The same authors also concluded that *An. coluzzii* prefers urban water collections and adapts quickly to pollution. These results corroborate those obtained by Djogbénou et al. [27]. The absence of *An. gambiae* in the agro-ecological zone of the far north could be due to the small number of surveyed localities in the only district of Malanville or to the physico-chemical properties of the breeding sites from which larvae were sampled [15].

In addition, only four individuals of *An. arabiensis* in Dassa, a savanna species previously found in northern Benin [27]. According to Djogbénou et al. [27] and Gnanguènon et al. [30], *An. arabiensis* would have moved to the centre of the country because of drought and human activities (destruction of forests concomitantly with the use of large areas for agriculture, hunting, grazing and timber harvesting and urbanization). Thus, the fact of finding it in the centre of the country (Dassa) testifies to its adaptation to the new climatic conditions, an evolutionary capacity peculiar to living beings. Although the effects of climate change such as drought and rain are not yet clearly quantifiable, it could be one of the causes of the displacement of living organisms. Insects are one of the best indicators to better appreciate the phenomenon. Species migration, such as the hypothesis given in the case of *An. arabiensis* for the present study, may contribute to the spread of this vector in Central and Southern Benin.

Considering the different surveyed zones, no significant difference was noted between the frequencies of the resistant allele *L1014F* of the *kdr* gene in the populations of *An. gambiae* and *An. coluzzii* (p > 0.05) except for those the central cotton and bar land zones (p < 0.05). Benin is a country where the most practiced activity is agriculture with similar agricultural practices (the use of pesticides and chemical fertilizers) from one locality to another. This could contribute to the wide distribution of the *L1014F* locus. Also, the climatic conditions of the laterite and central cotton agro-ecological zones are close or even identical. On the other hand, these two zones do not share the same climatic conditions as the agro-ecological zones of the north (far north of Benin, food crop producing of South-Borgou and Western Atacora) which are themselves close or even identical. This could also be a justifying argument. According to Akogbéto et al. [31] and Yadouléton et al. [9, 12], agricultural practices constitute a pressure of selection which spread the resistance allele in Benin. National coverage of insecticide-treated mosquito nets in the country could also contribute to the selection of the *L1014F* resistant allele. The presence and attachment of the *L1014F* allele of the *kdr* gene in some populations of *An. coluzzii*, which was not the case a few years ago [25] are evolutionary realities in our study area. This could possibly be generalized very soon. This is proof of the increase of the receptive potential the *L1014F* allele of the *kdr* gene in *An. gambiaes.l*. And certainly by genetic self-stop of the expansion of resistance genes in Benin. With this spectrum of expression of the resistance gene, It is inevitably moving towards a lack of effectiveness of the insecticide-based tools currently used in the fight against malaria vectors. The presence of this allele in *An. coluzzii* differs from one author to another, and several have reported its presence only in *An. gambiae* [25, 32]. The existence of the *L1014F* allele of the *kdr* gene in *An. coluzzii* could also be due to an ancestral phylogenetic inheritance or introgression process within its populations [33, 34], or to the migration of *An. coluzzii* carrying the resistant allele *kdr* "*L1014F*" from one locality to another [35] given the proximity of some of our areas. These results corroborate those obtained in Benin, Burkina Faso [36] and Mali [37]. The *L1014F* allele of the *kdr* gene is not directly distributed according to geography, but the ecology of the vectors in question is an indispensable factor in the selection of the resistant allele of the gene [9, 12, 13, 26]. A study of the genetic structure of these populations is necessary in order to explore their genetic dynamics in the different agro-ecological zones.

## Conclusion

In summary, for the moment that even if the data are in small portions, the *An*. *Arabiensis* species was found only in central Benin and the *L1014F* allele of the *kdr* gene is widespread and seems to fix in all the species recorded in the different agro-ecological zones. This situation amplifies the problem of resistance, which could eventually be a significant obstacle for the malaria vectors control. Similarly, a study of their genetic structure via the *L1014F* allele is necessary in order to put in place strategies to manage this resistance. These strategies will take into account both the ecology and the genetic diversity of the organisms involved to preserve the effectiveness of pyrethroids, the only insecticides used for the impregnation of mosquito nets.

## Data Availability

The data supporting the conclusions of this article are included in the article. Raw data will be available upon request to the corresponding author.

## Abbreviations

CREC: Cotonou Entomological Research Center
PNLP: Programme National de Lutte contre le Paludisme
KDR: knockdown resistance
WHO: Word Health Organization
